# Long-term repeatability and predictability of body temperature in dairy calves explored using reticulorumen temperature boluses

**DOI:** 10.1038/s41598-026-53703-0

**Published:** 2026-05-29

**Authors:** A. Jolivald, J. Vázquez-Diosdado, J. Kaler

**Affiliations:** 1 School of Veterinary Medicine and Science, University of Nottingham, Sutton Bonington Campus, Leicestershire, LE12 5RD UK; 2 School of Animal, Rural and Environmental Science, Nottingham Trent University, Brackenhurst Campus, Southwell Nottinghamshire, NG25 0QF UK

**Keywords:** Ecology, Ecology, Zoology

## Abstract

Research on the repeatability, plasticity and predictability of the body temperature of endotherms is limited. Following developments in precision livestock farming, dairy calves have emerged as a model organism to explore these questions in a large endotherm. This study aimed to harness long-term, high-frequency recordings from reticulorumen temperature boluses recordings to investigate the repeatability, plasticity and predictability of reticulorumen temperature before, during and after weaning in 98 dairy calves. Using both frequentist and Bayesian mixed modelling, we found that reticulorumen temperature was moderately repeatable (*R* = 0.258), that reticulorumen temperature was plastic over the three weaning stages but that plasticity varied on an individual level, and that individual calves differed in the predictability of their reticulorumen temperature (CV_P_ = 0.242). We also showed that weaning had a complex impact on reticulorumen temperature, its repeatability, and its predictability, with individual calves responding to weaning differently. This study is the first to formally quantify inter- and intra-individual variation in reticulorumen temperature in calves, adding to a restricted body of work on variation in body temperature in endotherms. We suggest accounting for inter- and intra-individual variation in methods aiming to automatically detect disease in calves using reticulorumen temperature would increase their accuracy.

## Introduction

Recently, variance partitioning techniques such as linear mixed models (LMMs) and double hierarchical generalised mixed models (DHGLMs) have allowed ecologists to objectively quantify the repeatability (inter-individual variance), plasticity and predictability (intra-individual variance) in trait expression^1–3^. While the repeatability^4–6^ and predictability^7–9^ of behavioural traits have been described extensively, the literature on individual differences in physiological traits remains more limited^10^. Traditionally, physiological research has focussed on describing a population mean or normal range for traits under exploration, with residual variation perceived as experimental noise^11^. Nonetheless, meaningful individual variation has since been documented in physiological traits such as whole animal metabolic rates^12,13^ and hormone levels^13,14^. Overall, these meta-analyses suggest that physiological traits have moderate repeatability, with mean estimates ranging from 0.42 for metabolic rate^12^ to 0.150 for hormone levels^13^. Notably however, there is substantial heterogeneity in repeatability estimates reported in individual experimental studies, suggesting a strong impact of experimental design on the repeatability estimates obtained. Meta-analyses suggest a significant impact of time between measurements^12–14^ and of context^14^ on repeatability.

While inter-individual variation in other physiological traits has received increased research attention in the last decade, research on the inter- and intra- individual variation in the body temperature of endotherms remains very limited. Most studies available have been conducted in laboratory rodents^15–17^, and body temperature is measured using a wide range of methods including the use of a rectal thermometer^17,18^, infra-red thermography^15^, and thermal radio-frequency identification implants^19^. Perhaps due to these differences in study species, experimental design and temperature measurement methods, there is wide variation in the repeatability estimates reported, ranging from 0. 12 (infra-red thermography in laboratory rodents^15^ to 0.92 (thermal radio-frequency identification implants in male passerine birds^19^. Therefore, additional research is needed in order to determine whether body temperature is a repeatable parameter, and which body temperature measurement methods may be best suited to estimating the repeatability of this physiological trait. It is notable that the highest repeatability estimates were obtained using automated, high-frequency measurement methods which allowed temperature data collection without interference with the subjects^19^. In addition to the repeatability of body temperature in tree swallows, Tapper et al.^19^ demonstrate population level and individual differences in plasticity in body temperature in response to ambient temperature. However, to the author’s knowledge this is the only study formally addressing individual differences in plasticity of body temperature. In addition, no study addresses predictability or intra- individual variations in body temperature. Therefore, while limited evidence is available on the repeatability of body temperature, additional research is needed to robustly document patterns of inter- and intra- individual variation in body temperature in endotherms.

Current advances in sensor technologies to study the repeatability, plasticity and predictability of body temperature in a larger endotherm under non-laboratory conditions. Given the large scale of the dairy industry, dairy calves are widely available, and calves of common dairy breeds, such as Holstein-Friesians, are kept under a wide range of climates^20^. Further, recent developments in precision livestock farming have seen the commercialisation of calf-specific indwelling temperature sensors designed to sit in the rumen (thereafter, reticulorumen temperature boluses), which automatically transmit reticulorumen temperature data^21,22^. These devices allow the collection of long-term, high frequency reticulorumen temperature data via telemetry, and thus are unaffected by potential impacts of human interaction and stress-induced hyperthermia^18,23,24^. Finally, pre-weaned calves function as monogastric animals, with milk intake bypassing the un-developed rumen and digested in the abomasum^25,26^. As calves age and weaning progresses, solid feed intake stimulates rumen development and the start of rumen fermentation, until calves become full ruminants after weaning^26^. Thus, characteristics of young calves’ digestive physiology enable the exploration of questions around the developmental and exogenous plasticity of reticulorumen temperature.

In addition to the fundamental relevance of adding to the currently limited body of work on inter- and intra-individual differences in body temperature in endotherms, research on patterns of variation in calves’ reticulorumen temperature is also of applied interest to the dairy industry. Reticulorumen temperature boluses are being used to detect pyrexia^21^ and bovine respiratory disease^22^ in calves using a fixed temperature threshold, so far with limited success. Understanding individual differences in body temperature could lead to the development of detection methods of pyrexia based on individualised thresholds.

This study aimed to investigate the long-term repeatability, plasticity and predictability of reticulorumen temperature in dairy calves by harnessing automated measures of reticulorumen temperature collected via reticulorumen temperature boluses. In line with the range of repeatability estimates reported for other physiological traits^12–14^, we hypothesized that reticulorumen temperature would show moderate repeatability. Due to the lack of pre-existing research on the plasticity and predictability of reticulorumen temperature, these analyses were purely exploratory. However, given the increase in reticulorumen function and drinking behaviour during weaning in calves^27^, we hypothesized that intra-individual variance in reticulorumen temperature would increase across the different weaning stages.

## Methods

### Ethical approval

The study was approved by the Ethics Committee of the School of Veterinary Medicine and Science, University of Nottingham (unique reference number 1481150603). All methods were performed in accordance with the relevant guidelines and regulations and are reported in accordance with the ARRIVE guidelines^28^.

### Subjects

98 female Holstein Friesian calves, split between 7 rearing groups (thereafter: cohorts; *n* = 12 to 16 calves), were recruited in the study between the 25th of March, 2022 and 13th of March 2023. It has been shown that more than 1000 observations and more than 20 individuals sampled provides reliable estimates for repeatability and intercept-slope correlations^29^. Hence, this study fulfils these requirements as they were 7994 observations and 98 animals. All procedures were carried out at the University of Nottingham’s Centre for Dairy Science Innovation (CDSI; Leicestershire, UK). The CDSI is a working dairy farm; all calves recruited in the study were bred for the purpose of dairy herd replacement and managed according to CDSI standard operating procedures and were housed indoors. Briefly, calves were removed from the dam within four hours of birth, fed 4 L of pasteurised colostrum and moved to paired housing in a 3 m x 2 m straw bedded pen. Routine calf husbandry procedures, including vaccinations to prevent bovine respiratory disease (BRD) and disbudding, were carried out while calves were in the paired housing. Calves remained in paired housing until a full cohort had been recruited, and the youngest calf in the cohort reached 21 days of age. The whole cohort was then moved to a 6 m x 10 m straw-bedded group-housing pen. Calves remained in the group housing for seven to ten weeks, during which they underwent a gradual weaning process, then moved to a heifer building following the end of the weaning process. All data used in this study was collected while the calves were in the group housing.

Calves were fed milk replacer (Milkivit Energizer ECM, Trouw Nutrition GB) in both the pair and the group housing using computerised milk feeding stations (Forster-Technik Compact Smart) from 2 days old until the end of weaning, with one teat per cohort available in group housing. The automatic milk feeder mixed a portion of milk replacer from milk powder and warm water (130 g/l) to feed the calf. In the pair housing allowance of milk replacer starts from 6 L at 2 days of age and increased gradually until reaching 10 L at 40 days of age. Daily milk allowance was also 10 L per calf for the first 35 days in group housing. A gradual step-down weaning procedure was then followed over the next 25 days, with the daily milk allowance reduced by 400mL a day until it reached 0 L (end of weaning process) by day 60 in group housing. In addition to milk replacer, chopped straw, calf starter concentrate feed and fresh water were available *ad libitum* in both paired and group housing.

In the group housing, calves were screened for BRD twice a week by trained research personnel. BRD diagnosis was made using a modified version of the Wisconsin score, based on respiratory symptoms and rectal body temperature^30^. A calf was considered to be sick on the day if they had a Wisconsin score of 5 or above and/or a rectal temperature of 39.5 °C or above, ad/or had been treated for illness on that day. Animals identified as sick was flagged to farm personnel and treated. In addition, in both housing, calves were regularly monitored by farm personnel, and received treatment as needed for diseases including BRD and calf diarrhoea. All treatments were recorded, providing records of healthy and sick days for each individual. There was a total of 178 day-instances out of 1365 day-instances (13.05%) that were removed due to calves scored as sick or treated. At the end of the study period the calves stayed at the CDSI and continued to follow the regular farm management protocols.

### Reticulorumen temperature measures

Reticulorumen temperature was measured in the calves using reticulorumen temperature boluses specifically designed for use in pre-weaned calves (FarmFit Bolus System, ST Genetics, USA) with an average error of $\:\pm\:0.2\:$°C. Following the manufacturer’s instructions, boluses were administered to study calves from a minimum of five days old, using a small ruminant bolus gun and did not require anaesthesia. The average age for bolus administration was 6.34 days. Once administered, boluses transmitted reticulorumen temperatures readings (°C) to a cloud-based storage every 15 min. The bolus’ battery life was expected to outlast the duration of the study and data was collected continuously until either the bolus malfunctioned, or calves were moved out of the group housing to the heifer building. The manufacturer’s online platform was used to monitor data flow on a weekly basis.

Raw reticulorumen temperature data was retrieved from the manufacturer’s systems and merged for pre-processing in R^31^. Water intake has been shown to strongly impact reticulorumen temperature in adult cows, resulting in outliers in reticulorumen temperature time series^32,33^. While water intake is limited in pre-weaned calves, it rapidly increases during and after weaning^27^ and may therefore impact reticulorumen temperature at this stage. In addition, outliers in reticulorumen temperatures time series may occur due to sensor errors^34^. Therefore, reticulorumen temperature data was pre-processed following previously published procedures^33^ to limit the impact of those outliers. Temperature readings lower than 35.6 °C or higher than 42.2 °C were filtered out as they likely corresponded to sensor errors^34^. Following this, a daily moving average and standard deviation of temperatures over the past four days were calculated for each day and calf. Any temperature lower than the daily moving average minus 3 moving standard deviations were then filtered out of the dataset^34^. Four days, rather than two weeks as in Bewley et al.^33^, were chosen as the moving average window duration to account for the significant impact of age on reticulorumen temperature in pre-weaned calves^21^, which is not present in adult cattle. Finally, for each calf, hourly mean temperatures were calculated using all retained datapoints.

### Ambient condition data

Ambient temperature and humidity conditions have been shown to significantly impact reticulorumen temperature in adult cattle^35^. Therefore, ambient temperature (°C) and humidity (%) data were collected on the CDSI for the duration of the study, using temperature loggers (ALTA 900 MHz Industrial Humidity Sensors with Probes). Ambient conditions were recorded every two hours. For each data point, the Temperature-Humidity Index (THI) was calculated according to NRC (1971), as cited by Mbuthia et al.^36^:

$$THI{{}} = {{}}\left( {1.8\:\:{{\times}}\:\:Temperature{{}} + {{}}32} \right){{}}-{{}}\left({0.55{{}}-{{}}0.0055\:\:{{\times}}\:\:Humidity}\right){{\times}}\left({1.8{{}}\:\:{\times}\:\:{{}}Temperature{{}}-{{}}26}\right)$$

Linear interpolation was then used in R (*approxfun()* function to approximate temperature, humidity and THI data points at hourly intervals, to match the bolus data resolution. Ambient temperature ranged from − 6.45 °C to 36.27 °C with an average 12.30 °C 

$\:(\pm\:6.72^\circ\:\mathrm{C}$) during this study, whereas THI ranged from 20.38 to 82.35 with an average 53.63($\:\pm\:$10.90). During this period there were no times when THI was above was 90(severe heat stress) and 0.60% of the time was between 80 and 90 (moderate heat stress) and 2.92% was between 72 and 80 (mild heat stress).

### Data analysis

#### Data selection

For each calf, reticulorumen temperature data was extracted only on days on which the calf had been health scored and they were labelled as healthy (Wisconsin score < 5 and rectal temperature below 39.5 °C) by research personnel and had not received any treatment from farm personnel.

In line with the significant impact of time of day on reticulorumen temperature in adult cattle^35,37^, our calf reticulorumen temperature data showed a strong circadian rhythm at all stages of weaning. Therefore, the dataset was further restricted to data points collected between 9am and 5pm, when changes in reticulorumen temperature were minimal and largely linear, to satisfy the assumptions of the model.

For each data point, the calf ID number and age in days, date and time of measurement and corresponding THI, as well as weaning status (Pre-Weaned, Step-Down, Weaned), were included in the dataset. Any days for which THI information was not available were subsequently excluded from the dataset.

#### Data analyses

All statistical analysis was carried out in R v.4.2.3^31^ using code adapted from Hertel et al.^38^.

##### Repeatability of reticulorumen temperature

The repeatability of reticulorumen temperature on all healthy days (*N* = 98 calves) was assessed using a Gaussian linear mixed model (LMM)^1^ fitted using the ***lme4*** R package^39^ and formulated as follows:

2.1 $$\:\begin{array}{c}{Y}_{ijkl}=\left({\beta\:}_{0}+{ind}_{{0}_{j}}+{coh}_{{0}_{k}}+{dat}_{{0}_{l}}\right)+\:{\beta\:}_{1}{X}_{{1}_{ijkl}}+\:{\beta\:}_{2}{X}_{{2}_{ijkl}}+\:{\beta\:}_{3}{X}_{{3}_{ijkl}}+\:{\beta\:}_{4}{X}_{{4}_{ijkl}}+{e}_{{0}_{ijkl}}\:\end{array}$$

2.2 $$\:\begin{array}{c}\left[{ind}_{{0}_{j}}\right]\:\sim\:N\left(0,\:{\varOmega\:}_{ind}\right):\:{\varOmega\:}_{ind}=\left[{V}_{{ind}_{0}}\right]\end{array}$$

2.3 $$\:\begin{array}{c}\left[{coh}_{{0}_{k}}\right]\:\sim\:N\left(0,\:{\varOmega\:}_{coh}\right):\:{\varOmega\:}_{coh}=\left[{V}_{{coh}_{0}}\right]\:\end{array}$$

2.4 $$\:\begin{array}{c}\left[{dat}_{{0}_{l}}\right]\:\sim\:N\left(0,\:{\varOmega\:}_{dat}\right):\:{\varOmega\:}_{dat}=\left[{V}_{{dat}_{0}}\right]\end{array}\:$$

2.5 $$\:\begin{array}{c}\left[{e}_{{0}_{ijkl}}\right]\:\sim\:N\left(0,\:{\varOmega\:}_{e}\right):\:{\varOmega\:}_{e}=\left[{V}_{{e}_{0}}\right]\end{array}$$

In this equation, $\:{Y}_{ijkl}$ represents the *ith* reticulorumen temperature measure collected for individual *j*, from cohort *k*, on date *l*. β_0_ represents the grand mean value of reticulorumen temperature, and $\:{ind}_{{0}_{j}},\:{coh}_{{0}_{k}}$and $\:{dat}_{{0}_{l}}$ are random intercepts associated with individual *j*, cohort *k* and date *l*, respectively. $\:{\beta\:}_{1}{X}_{{1}_{ijkl}},\:\:{\beta\:}_{2}{X}_{{2}_{ijkl}},\:\:{\beta\:}_{3}{X}_{{3}_{ijkl}}$ and $\:{\beta\:}_{4}{X}_{{4}_{ijkl}}$ represent the fixed effects of age in days, THI, weaning status and time of day, respectively. Age and THI were entered into the model as continuous predictors, while weaning status and time of day, rounded to the nearest hour, were entered as factors.

To assess whether repeatability of reticulorumen temperature changed between each stage of weaning, three additional LMMs were fitted to subsets of the data comprising measures at the pre-weaning stage only (*N* = 82 calves), at the step-down stage only (*N* = 80 calves), and at the weaned stage only (*N* = 59 calves), respectively. Models were formulated as above (Eq. 2.1 to 2.5), except weaning stage was not included as a fixed effect.

For all LMMs, the repeatability of reticulorumen temperature was calculated as the proportion of total variance in each dataset explained by individual calf identity, once fixed and random effects were taken into account^1^:

2.6 $$\:\begin{array}{c}R=\:\frac{{V}_{ind}}{{V}_{ind}+\:{V}_{coh}+\:{V}_{dat}+\:{V}_{e0}}\:\end{array}$$

Simulated runs (*n* = 1000) of each model were then used to generate a posterior distribution of variance components and calculate credible intervals for repeatability, as well as best linear unbiased predictors (BLUP) of reticulorumen temperature for each calf (Hertel et al., 2020).

##### Plasticity of reticulorumen temperature

Individual plasticity in reticulorumen temperature across the three weaning stages was assessed using a LMM with random slopes for weaning stages^2^, fitted using the ***lme4*** R package^39^ and formulated as follows:

2.7 $$\:{Y}_{ijkl}=\left({\beta\:}_{0}+{ind}_{{0}_{j}}+{coh}_{{0}_{k}}+{dat}_{{0}_{l}}\right)+\:{\beta\:}_{1}{X}_{{1}_{ijkl}}+\:{\beta\:}_{2}{X}_{{2}_{ijkl}}+\:{(\beta\:}_{3}+{u}_{{3}_{j}}){X}_{{3}_{ijkl}}+\:{\beta\:}_{4}{X}_{{4}_{ijkl}}+{e}_{{0}_{ijkl}}\:$$

2.8 $$\:\begin{array}{c}\left[{ind}_{{0}_{j}}\right]\:\sim\:N\left(0,\:{\varOmega\:}_{ind}\right):\:{\varOmega\:}_{ind}=\left[{V}_{{ind}_{0}}\right]\end{array}$$

2.9 $$\:\begin{array}{c}\left[{coh}_{{0}_{k}}\right]\:\sim\:N\left(0,\:{\varOmega\:}_{coh}\right):\:{\varOmega\:}_{coh}=\left[{V}_{{coh}_{0}}\right]\end{array}\:$$

2.10 $$\:\begin{array}{c}\left[{dat}_{{0}_{l}}\right]\:\sim\:N\left(0,\:{\varOmega\:}_{dat}\right):\:{\varOmega\:}_{dat}=\left[{V}_{{dat}_{0}}\right]\end{array}$$

2.11 $$\:\begin{array}{c}\left[{e}_{{0}_{ijkl}}\right]\:\sim\:N\left(0,\:{\varOmega\:}_{e}\right):\:{\varOmega\:}_{e}=\left[{V}_{{e}_{0}}\right]\end{array}$$

Where $\:{u}_{{3}_{j}}$ is a random slope representing individual variations in slope associated the successive weaning stages, and all other notations are identical to the model described in 2.5.2.2. This analysis was restricted to calves who had data available at all three stages of weaning (*N* = 44) to facilitate model convergence (Hertel et al., 2020). Reaction norms were used to investigate the plasticity of reticulorumen temperature in response to successive weaning stages^2^. In addition, intercept-slope correlations were used to investigate whether individuals differed in their responses to changes in weaning stages^2^.

##### Predictability of reticulorumen temperature

A Double Hierarchical Generalised Linear Model (DHGLM) was used to investigate individual variation in the predictability of reticulorumen temperature on all healthy days to investigate individual variation in the predictability of reticulorumen temperature^3^. In addition to a mean part common to other linear models, DHGLMs also comprise a dispersion part, which allows modelling of the residual variance to estimate between-individual variation in intraindividual variance^3^. The model was formulated as follows:

2.12 $$\:\begin{array}{c}{Y}_{i}=\:X\beta\:+Z\alpha\:+\:\varepsilon\:\:\end{array}$$

2.13 $$\:\begin{array}{c}\alpha\:\:\sim\:N\left(0,\:{I}_{m}{\sigma\:}_{\alpha\:}^{2}\right),\:\end{array}$$

2.14 $$\:\begin{array}{c}\varepsilon\:\:\sim\:N\left(0,\:Diag\left\{{\sigma\:}_{\varepsilon\:}^{2}\right\}\right),\:\end{array}$$

2.15 $$\:\begin{array}{c}\mathrm{log}\left({\sigma\:}_{\varepsilon\:}\right)=\:{\eta\:}_{d},\:\end{array}$$

2.16 $$\:\begin{array}{c}{\eta\:}_{d}=\:{Z}_{d}{\alpha\:}_{d},\:\end{array}$$

2.17 $$\:\begin{array}{c}{\alpha\:}_{d}\:\sim\:N\left(0,\:{I}_{m}{\omega\:}_{{\sigma\:}_{d}}^{2}\right)\:\end{array}$$

In those equations $\:{Y}_{i}$ represent the *ith* reticulorumen temperature measure, *X* the fixed effects of the mean part of the model, *Z* and *Z*_*d*_ the random effects of the mean and dispersion parts of the model, respectively, and $\:\varepsilon\:$ the residual variance. $\:{\sigma\:}_{\varepsilon\:}^{2}$ represents individual-specific residual standard deviations, which are assumed to follow a log-normal distribution, and $\:{\omega\:}_{{\sigma\:}_{d}}^{2}$ is the dispersion model hyperparameter^3^. In the mean part of the model, age in days, THI, weaning stage and time of day (as a factor) were used as fixed effects while calf ID number, cohort and date were used as random effects. No fixed effects, and only calf ID number as random effect, were used in the dispersion part of the model.

To assess whether predictability of reticulorumen temperature changed between each stage of weaning, three additional DHGLMs were fitted to subsets of the data comprising measures at the pre-weaning stage only (*N* = 82 calves), at the step-down stage only (*N* = 80 calves), and at the weaned stage only (*N* = 59 calves), respectively. Models were formulated as above (Eq. 2.12 to 2.17), except weaning stage was not included as a fixed effect for the mean part of the model.

All DHGLMs were fitted using the ***brms*** package in R^40^, using uninformative priors for both fixed and random effects, 4 chains of 15 000 iterations including 7 500 warm-up iterations, and a thinning interval of 1.

Individual differences in predictability in the sample calves were assessed by extracting $\:{\omega\:}_{{\sigma\:}_{d}}^{2}$, the dispersion model hyperparameter (estimate for individual differences in residual variance) and 95% confidence interval from each DHGLM. In addition, a population-level estimate of variation in predictability was calculated as follows, after Cleasby et al.^3^:

2.18 $$\:\begin{array}{c}{CV}_{P}=\:\sqrt{\left(\mathrm{exp}\left({\omega\:}^{2}-1\right)\right)}\:\end{array}$$

## Results

### Impact of fixed predictors on hourly reticulorumen temperature

Time of day had a strong, significant impact on hourly reticulorumen temperature, with hourly temperature significantly increasing over time between 9am and 5pm (Table 1). In addition, there was a weak (0.069 ($\:\pm\:0.032$), Table 1) but significant increase in reticulorumen temperature post-weaning (t = 2.14, df = 1595.449, *p* = 0.032). Age had a very small and not significant effect (0.001 $\:\pm\:0.001$) on reticulorumen temperature (t = 1.748, df = 293.119, *p* = 0.082) However, there was no significant impact of THI on reticulorumen temperature. Overall, the fixed effects considered here explained only a very small proportion of total variance in rumen temperature (R^2^_m_ = 0.051), although the full model including random effect explained approximately 40% of variance in the dataset (R^2^_c_ = 0.401).

### Repeatability of reticulorumen temperature

**Fig. 1:**
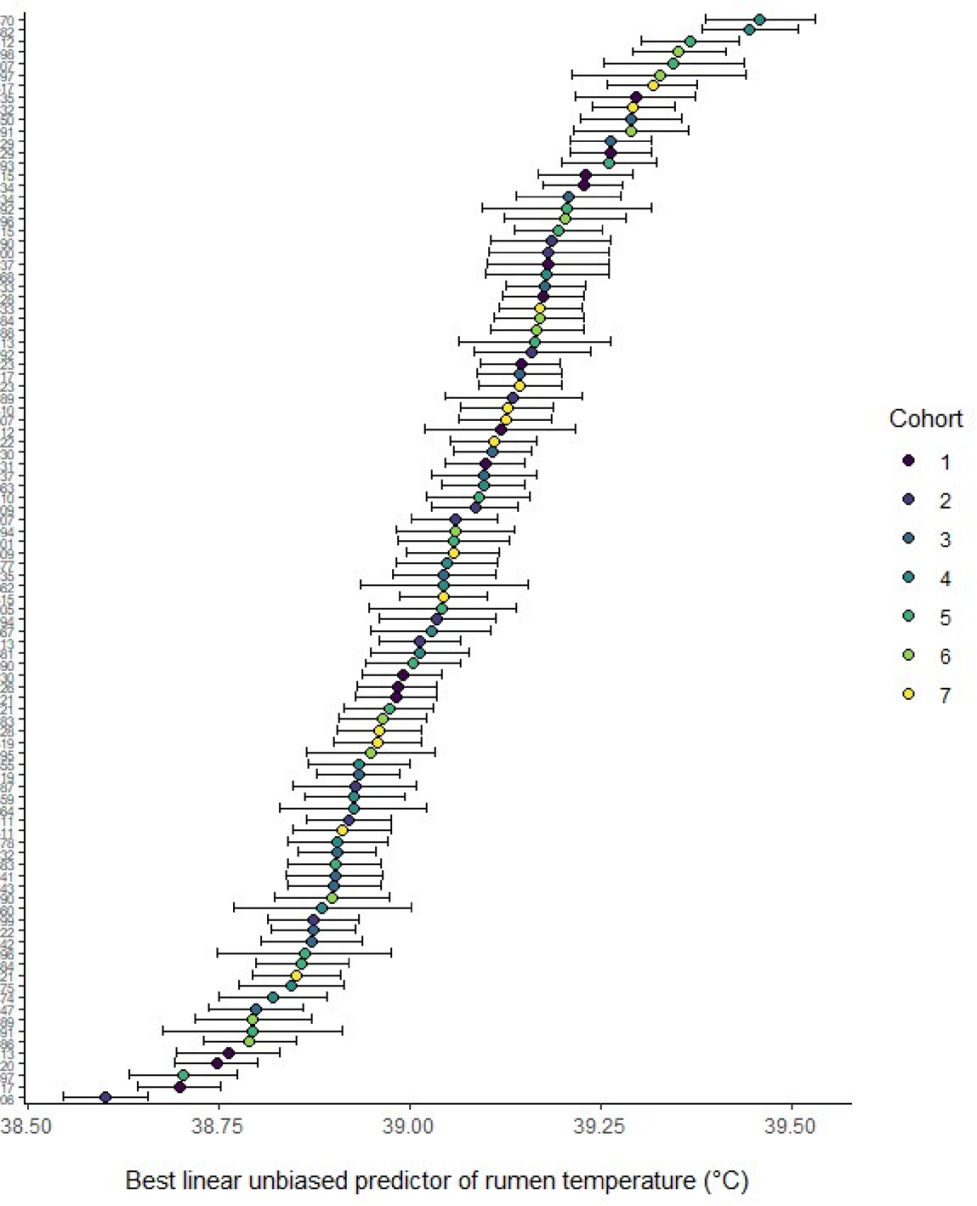
Best Linear Unbiased Predictors (BLUPs) of reticulorumen temperature (°C) for the *N* = 98 study calves (*n* = 7994 data points). Individual calves are sorted in increasing order of BLUP and identified by their ID number on the y axis.

The adjusted repeatability of reticulorumen temperature on all healthy days (*n* = 98 calves) was 0.248. The repeatability estimate and 95% credible interval derived from simulated model runs was 0.258 [0.234–0.285]. BLUPs of reticulorumen temperature in the 98 study calves ranged from 38.60 ± 0.05 °C to 39.45 ± 0.072 °C (Fig. 1).

When each stage of weaning was considered separately, adjusted repeatability estimates were comparable pre-weaning (*n* = 82 calves; R_adj_ = 0.322, 95% CI = [0.299–0.380]) and during step-down weaning (*n* = 80 calves; R_adj_ = 0.316, 95% CI = [0.290–0379]), but lower post-weaning (*n* = 59 calves; R_adj_ = 0.225, 95% CI = [0.193–0.296]).

### Reaction norms of reticulorumen temperature across weaning stages

**Fig. 2:**
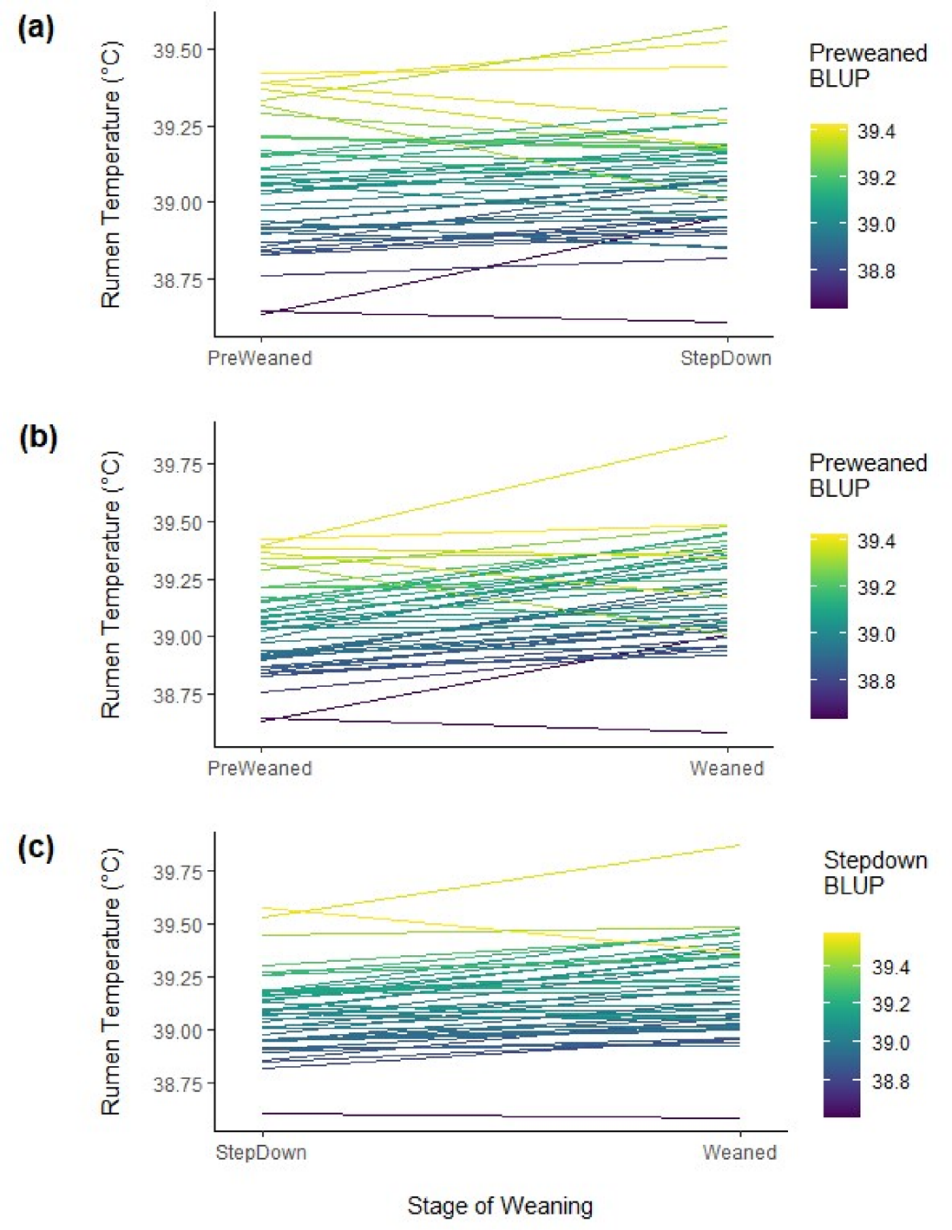
Reaction norms plots depicting intercept/slope correlations between **(a)** pre-weaning and step-down weaning; **(b)** pre- and post-weaning; and **(c)** step-down and post-weaning stages. Lines represent individual calves and are coloured in order of intercept values to improve readability of trajectories for different intercept values.

There was significant individual plasticity in reticulorumen temperature, with individuals varying in both elevation and slope of reticulorumen temperature across the three weaning stages (Fig. 2). There was a significant negative intercept-slope correlation between the pre-weaning and step-down stages (*r* = −0.38, *p* = 0.011), i.e. calves with a higher reticulorumen temperature pre-weaning underwent less of an increase in reticulorumen temperature during step-down compared to calves with a lower pre-weaning reticulorumen temperature (Fig. 2a). By contrast, there was a significant positive intercept-slope correlation between the step-down and post-weaning stage (*r* = 0.68, *p* < 0.001), i.e. calves with a higher reticulorumen temperature during step-down weaning underwent a larger reticulorumen temperature increase post-weaning (Fig. 2b). Finally, there was no significant intercept-slope correlation between the pre- and post-weaning stage (*r* = −0.22, *p* = 0.215; Fig. 2c).

### Predictability of reticulorumen temperature

**Fig. 3:**
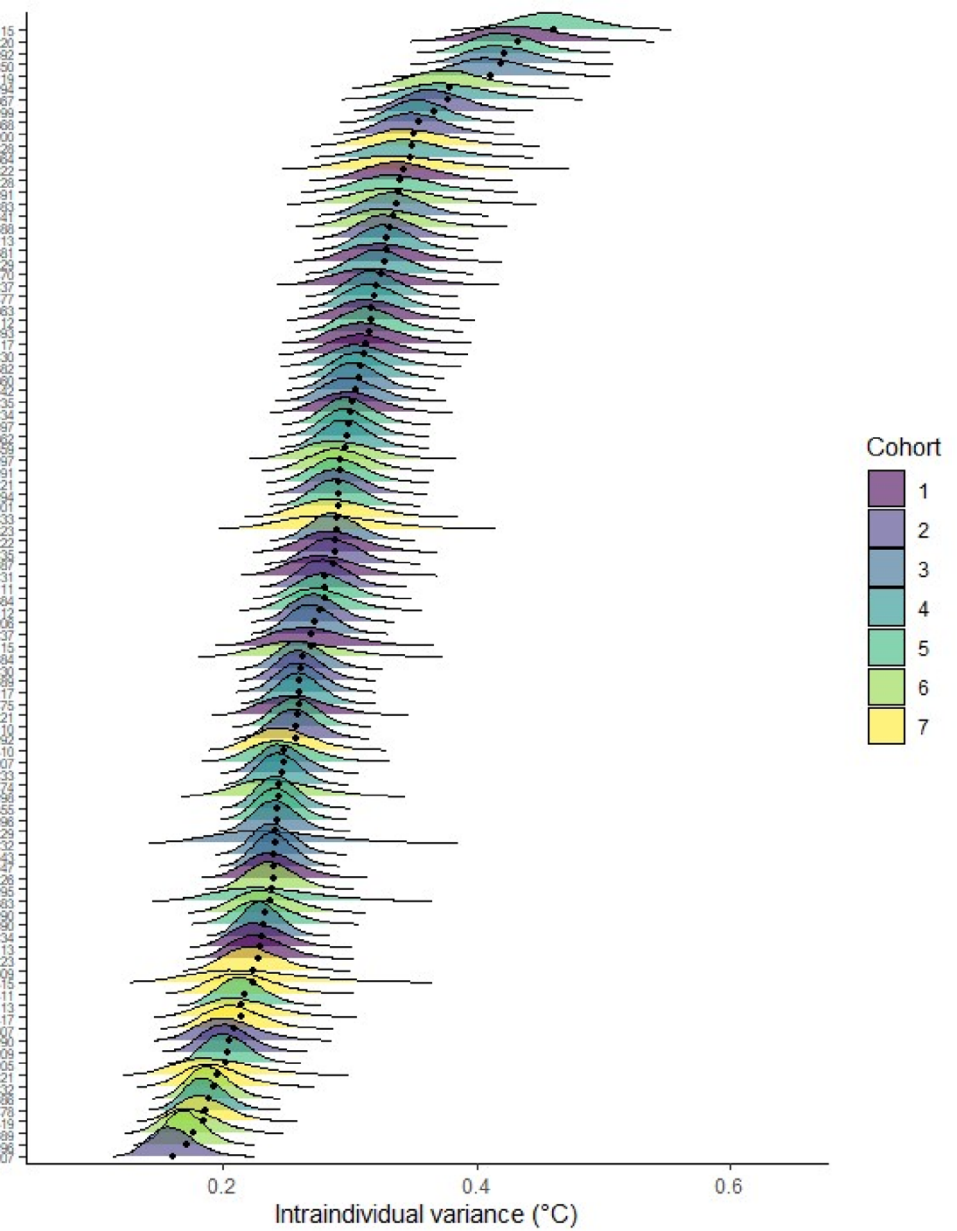
Back transformed posterior distributions of residual intraindividual variation (rIIV) from the double hierarchical mixed models for reticulorumen temperature (°C) for each calf with healthy days at all stages of weaning included in the analysis (*n* = 7994 data points; *N* = 98 calves). The ridges indicate the posterior 95% credible interval, and the different colours correspond to the different cohorts.

Calves varied in the predictability of their reticulorumen temperature (Fig. 3). When healthy days at all stages of weaning were entered into the analysis, the dispersion model hyperparameter (estimate of individual differences in residual variance) and 95% confidence interval extracted from the DHGLM was 0.239 [0.201–0.283]. The coefficient of variation in predictability CV_P_ was 0.242 [0.202–0.287].

Separate analyses for each weaning stage showed that individual predictability decreased as weaning progressed, with backtransformed estimates of residual intra-individual variance ranging from 0.120 to 0.552 °C pre-weaning, from 0.149 to 0.425 °C during stepdown weaning, and from 0.299 to 0.487 °C post-weaning. However, inter-individual variation in predictability also decreased as weaning progressed (pre-weaned: CV_P_ = 0.346 [0.285–0.411]; step-down weaning: CV_P_ = 0.282 [0.226–0.347]; post-weaning: CVP = 0.158 [0.104–0.213]).

**Table 1:** Summary of a linear mixed model (R^2^_m_ = 0.051; R^2^_c_ = 0.401) of rumen temperature on all healthy days (*n* = 7994 data points; *N* = 98 calves).

	Estimate	Std. Error	t value	df	*p* value
(Intercept)	39.051	0.095	409.381	57.746	< 0.001 ***
Age	0.001	0.001	1.748	293.119	0.082
THI	−0.002	0.001	−1.190	113.131	0.237
Weaning: Step-Down	−0.013	0.019	−0.663	3510.462	0.507
Weaning: Weaned	0.069	0.032	2.140	1595.449	0.032 *
Time 10:00	0.041	0.014	2.958	7857.014	0.003 **
Time 11:00	0.114	0.014	7.924	6666.640	< 0.001 ***
Time 12:00	0.116	0.015	7.735	4162.948	< 0.001 ***
Time 13:00	0.122	0.015	7.878	2472.182	< 0.001 ***
Time 14:00	0.077	0.016	4.781	1634.094	< 0.001 ***
Time 15:00	0.063	0.016	3.811	1267.846	< 0.001 ***
Time 16:00	0.166	0.017	10.005	1222.456	< 0.001 ***
Time 17:00	0.249	0.016	15.111	1396.082	< 0.001 ***

## Discussion

Our study is the first to document the impact of weaning on reticulorumen temperature in farmed dairy calves, with long-term data automatically collected using commercial precision livestock farming sensors to characterise long-term patterns of variation of reticulorumen temperature in calves and, more generally, to use telemetry to quantify inter- and intra-individual variations in body temperature in an endotherm. Our aim was to harness long-term, continuous recordings of rumen temperature obtained using reticulorumen temperature boluses to investigate the repeatability, plasticity and predictability of reticulorumen temperature before, during and after weaning in dairy calves. Using both frequentist and Bayesian mixed modelling approaches, we found that reticulorumen temperature was moderately repeatable, that there was clear population-level plasticity in reticulorumen temperature in response to the different weaning stages, but that plasticity varied on an individual level, and that individual calves differed in the predictability of their reticulorumen temperature. We also found that repeatability and inter-individual differences in plasticity decreased after weaning.

Our study has fundamental relevance to physiological ecology, as it is one of the first to quantify the long-term repeatability of body temperature in an endotherm, and the first to quantify the predictability of body temperature. Metabolic rate, i.e. the minimum rate of energy expenditure necessary for endotherms to maintain energy balance, including constant body temperature, in neutral conditions, is highly repeatable^12,41^ and emerging research suggests there is little inter-individual variation in its predictability^8^. By contrast, research on inter- and intra-individual differences in body temperature itself remains very limited in endotherms, perhaps due to its perception as a highly regulated, and thus stable, physiological variable^24^. Our repeatability analysis shows that individual calves differ in their typical reticulorumen temperature when healthy. The repeatability estimates derived from our study are comparable to those obtained from repeated rectal temperature measures in house mice (*Mus domesticus*)^17^, suggesting that the magnitude of inter-individual differences in body temperature is consistent across the two species. In addition, we demonstrated that calves’ reticulorumen temperature responds plastically to changes in the calves’ dietary provisions over the course of weaning.

Exogenous plasticity (as defined by Stamps^42^, and inter-individual differences in plasticity, in body temperature in response to changes in environmental conditions (ambient temperature and food availability) have been documented in tree swallows (*Tachycineta bicolor*)^19^ and yellow-necked mice (*Apodemus flavicollis*)^16^. In both species, this plasticity may be due to differences in thermoregulatory efficiency under different environmental conditions^16,19^. More research is needed to determine whether the changes observed in reticulorumen temperature over the stages of weaning similarly correspond to developmental plasticity, associated with reticulorumen maturation during weaning^26^, or whether they also represent exogenous plasticity in response to the change of environment weaning represents for the calves.

While fluctuations in body temperatures have previously been documented in healthy mammals (e.g. Gordon^24^, this study is the first to formally quantify intra-individual variations in body temperature in an endotherm. Our results demonstrate that individual calves vary in the predictability of their reticulorumen temperature. From an evolutionary point of view, this raises the question of whether, and how, predictability of reticulorumen temperature may affect fitness.

Our results on the repeatability and predictability of rumen temperature are of applied importance for the dairy industry. The repeatability estimate for reticulorumen temperature over all stages of weaning was 0.258 [0.234–0.285], i.e. over a quarter of total variation in calves’ reticulorumen temperature was explained by individual ID. In addition, calves’ typical reticulorumen temperature when healthy showed a wide range of variation throughout the day (0.86 °C). Finally, there were strong inter-individual differences in predictability in reticulorumen temperature (CV_P_ = 0.242 [0.202–0.287]), with some calves being more predictable in their reticulorumen temperature than others. Given the recent industry interest in utilising rumen temperature boluses to detect pyrexia^21^ or bovine respiratory disease^22^ in calves, as well as oestrus and ovulation in adult cows^43^, these results provide important insights into individual-specific characteristics of reticulorumen temperature, which may help refine detection methods. Indeed, the large inter-individual variations in typical reticulorumen temperature on healthy days may explain the limited success of methods utilising fixed reticulorumen temperature thresholds to detect pyrexia in calves^21^ or oestrus and ovulation in adults^43^. Our results suggest that the use of individual specific thresholds may be a more successful approach, in line with a previous study which detected drinking events more accurately based on cow-specific, rather than fixed, reticulorumen temperature thresholds^44^. More research is also needed to determine whether individual differences in reticulorumen temperature may impact welfare and/or performance.

While reticulorumen temperature has been studied in calves^21,22^, our study is the first to document the impact of weaning on reticulorumen temperature and its inter- and intra-individual variation in a young ruminant. At the population level, there was a significant increase in reticulorumen temperature post-weaning, as indicated by the significant effect of weaning detected by the linear mixed model used in the repeatability analysis, as well as the clear population-level plasticity in response to weaning visible on Fig. 2. Notably, the increase in reticulorumen temperature only became significant post-weaning, rather than during the stepdown weaning process. The model estimate suggests that reticulorumen temperature increased by 0.07 °C on average, from pre- to post-weaning. This increase is likely linked with an increase in reticulorumen development and fermentation associated with increased solid feed consumption, as calves transition from monogastric digestion in the abomasum pre-weaning to full rumination^26^.

Weaning was also associated with increased intra-individual variation in reticulorumen temperature. The repeatability of reticulorumen temperature significantly decreased post-weaning (*R* = 0.225 [0.193–0.296]) compared to pre-weaning (*R* = 0.322 [0.299–0.380]) and during stepdown (*R* = 0.316 [0.290–0379]), which indicates that the proportion of variance accounted for by intra-individual differences (residual variance in the linear mixed model) became higher post-weaning^1,3^. In accordance with this, the range of back-transformed estimates of residual intra-individual variance derived from the DHGLM also indicated that intra-individual variability increased over weaning stages. This increase in variability post weaning is likely linked with the digestive physiology of calves. Pre-weaning, calves function as pseudo-monogastric animals, as milk intake bypasses the rumen and is digested directly in the abomasum, and the rumen serves little digestive function^26^. Thus, intra-individual variability in reticulorumen temperature pre-weaning is likely to largely reflect intrinsic variability in body temperature. By contrast, in adult cows, water^32,33^ and solid feed^45^ intake have been shown to result in strong, short-term variations in reticulorumen temperature. The dietary changes associated with weaning are therefore likely to explain at least in part the post-weaning increase in intra-individual variability we document here, especially as individual differences have been observed in how early calves start eating solid feed^46,47^ and large individual variation in dietary choices at young age^48^. Thus, post-weaning, intra-individual variations in reticulorumen temperature likely reflect a combination of intrinsic variability in reticulorumen temperature, and variability linked with feed and water intake. Further work may be needed to tease apart the impact and relative importance of those two sources of variation.

In addition to these population level patterns, there were inter-individual differences in calves’ reticulorumen temperature responses to weaning. Figure 2 shows there were significant inter-individual differences in plasticity in response to weaning. Slope-intercept correlations varied in both strength and direction over the three stages of weaning, suggesting the relationship between reticulorumen temperature phenotype pre-weaning and the evolution of reticulorumen temperature during and after weaning is complex. Overall, individuals with extreme reticulorumen temperature pre-weaning experienced the strongest plasticity during weaning: some individuals with the highest pre-weaning BLUPs actually experienced a decrease in reticulorumen temperature post-weaning, while those with the lowest pre-weaning BLUPs experienced the largest increase. In addition, intra-individual variability did not increase to the same degree in all calves. The upper bounds of back-transformed estimates remained constant across weaning stages, indicating that highly variable individuals pre-weaning did not increase in variability. However, the lower bound of estimates increased over the weaning stages, indicating that individuals with more constant reticulorumen temperature pre-weaning became more variable as weaning progressed. This pattern is also confirmed by the decrease in inter-individual differences in intra-individual variability (CV_P_) as weaning progresses, which shows the population became more uniformly (highly) variable post-weaning. Repeatable inter-individual differences in total feed intake, meal frequency and feeding rate exist in both pre-weaned calves^7^ and adult cattle^49^. Calves also differ in the predictability of their milk intake behaviour^7^. Therefore, inter-individual differences in feed and water intake behaviour may drive some of the variation in reticulorumen temperature response to weaning we document here. In addition, *ad libitum* solid feed and water was always available to the calves in our sample, including pre-weaning. Calves who already exhibited higher reticulorumen temperature BLUPs and/or intra-individual variability pre-weaning may therefore be calves who had a higher voluntary solid feed intake prior to weaning.

Some limitations of this work must be acknowledged. First, our sample was highly homogenous, as it was limited to only one breed and sex of cattle, kept on a single farm and managed uniformly. Sex impacts the repeatability and plasticity of body temperature in tree swallows^19^, and management techniques such as the timing of weaning and the type of solid feed provided impacts the development of reticulorumen fermentation in young calves^26^. Additionally, the consumption of straw and started was not measured and it has been shown that diet has a significant impact on the amount of heat generated in the rumen^50^. Therefore, care should be taken in generalising our findings, and more research is needed to evaluate the impact of sex, breed, diet and management systems on the repeatability, plasticity and predictability of reticulorumen temperature in young ruminants.

In addition, calves were screened for disease by trained research personnel using the Wisconsin score^30^ in order to ensure only temperatures from healthy days were included in analysis. However, Wisconsin scores may have limited sensitivity and specificity in identifying bovine respiratory disease in calves^51,52^. Therefore, it is possible that data from diseased calves were accidentally included in our sample if the Wisconsin scoring system failed to detect illness.

Finally, although we conducted a thorough literature review to identify potential factors affecting reticulorumen temperature, the percentage of unexplained variance in our models remained very high, with the fixed effects explaining only 5.1% of total variation in our sample. Given the mathematical definition of repeatability, this may may have artificially lowered our repeatability estimate. Therefore, more work is needed to identify sources of variation in reticulorumen temperature in dairy calves.

In conclusion, our study showed that inter-individual differences make up a quarter of all variation in reticulorumen temperature in pre-weaned and weaned dairy calves, and that individual calves also vary in their predictability. We also showed that while reticulorumen temperature responds plastically to weaning at the population level, individual calves differ in their responses. These results are significant for the dairy industry, as accounting for those individual differences in predictive models of disease or oestrus may increase their accuracy. However, these results are also of fundamental importance, as our study was one of the first to quantify the long-term repeatability of reticulorumen temperature using telemetry in an endotherm, and it is the first to formally quantify intra-individual variation, and inter-individual differences in plasticity, of reticulorumen temperature in an endotherm.

## Data Availability

Data to reproduce the results in this study is available from the corresponding author upon reasonable request.
